# Traditional Transportation Methods and Their Influence on Local Chicken Welfare, Behavior, and Blood Profiles: A Policy Considerations

**DOI:** 10.3390/vetsci12090798

**Published:** 2025-08-23

**Authors:** Saber Y. Adam, Abdelkareem A. Ahmed, Mohammed H. Jammaa, Mohammed Rashid AL Makhmari, Hosameldeen Mohamed Husien, Mohamed Osman Abdalrahem Essa, Hamada Elwan, Mohamed Shehab-El-Deen, Shaaban S. Elnesr, Ahmed A. Saleh, Demin Cai

**Affiliations:** 1 College of Animal Science and Technology, Yangzhou University, Yangzhou 225009, China; saber@duc.edu.sd (S.Y.A.); elemlak1339@gmail.com (A.A.S.); 2 Biomedical Research Institute, Darfur University College, Nyala 155, Sudan; kareemo151@gmail.com; 3 College of Law and Sharia, University of Nyala, Nyala 155, Sudan; mtemsah@su.edu.om (M.H.J.); mmakhmari@su.edu.om (M.R.A.M.); 4 College of Veterinary Medicine, Albutana University, Rufaa 22217, Sudan; mohosman0999@gmail.com; 5 Animal and Poultry Production Department, Faculty of Agriculture, Minia University, El-Minya 61519, Egypt; hamadaelwan83@mu.edu.eg; 6 Department of Animal and Poultry Production, College of Agriculture and Food, Qassim University, Buraydah 52571, AL-Qassim, Saudi Arabia; m.shehabeldeen@qu.edu.sa; 7 Department of Poultry Production, Faculty of Agriculture, Fayoum University, Fayoum 63514, Egypt; ssn00@fayoum.edu.eg; 8 Animal and Fish Production Department, Faculty of Agriculture (Al-Shatby), Alexandria University, Alexandria 11865, Egypt

**Keywords:** animal welfare, chicken transportation, farmers, oxidative stress

## Abstract

Indigenous chickens are raised in various rural areas in large quantities throughout Sudan. They must be transported over various distances to centralized slaughterhouses or for other purposes. In this study, we examined indigenous chicken farmers’ perceptions of chicken welfare during transportation. A total of 160 indigenous chickens (80 control + 80 transported with their owners) participated in this study. Our findings revealed that 69% and 88% of the farmers indicated that they were not knowledgeable about animal rights and animal welfare, respectively. The majority of the farmers (86%) reported that they were unaware of animal protection laws. Furthermore, the transported chickens showed a significantly long tonic immobility duration (*p* < 0.05) compared to the control chickens. Moreover, low pecking behavior was significant (*p* < 0.05) in transported chickens compared to control, particularly on day one of the experiment. In addition, the mean values of glucose, TWBCs, monocytes, basophils, eosinophils, H/L ratio, Hb, MCHC, and PLT were significantly higher (*p* < 0.05) in transported chickens compared to the controls. In addition, TNF-a, IL-1β, IL-2, IL-4, IFN-γ, IL-17, as well as ROS, MDA, cortisol, glucose, and total cholesterol were significantly higher (*p* < 0.05) in transportation chickens compared to control, while CAT, GSH, ATP, and SOD were significantly lower (*p* < 0.05) in transportation chickens compared to control. We conclude that the traditional transportation of indigenous Sudanese chickens affected their welfare, and this was associated with farmers’ low perceptions of chicken welfare, and stress-induced blood profile changes.

## Simple Summary

This study evaluated farmers’ perceptions of chicken welfare, as well as the effects of transportation, on Sudanese native chickens. The majority of farmers were found to be ignorant about animal rights and welfare. In addition to displaying decreased pecking behavior, particularly on the first day, transported chickens showed considerably longer tonic immobility, which suggests increased stress. In addition to higher oxidative stress markers and inflammatory cytokines, blood examinations of transported chickens showed raised levels of glucose, total white blood cells, monocytes, basophils, eosinophils, hemoglobin, MCHC, and platelets. On the other hand, oxidative stress and lack of energy were indicated by lower levels of ATP and antioxidant enzymes. Generally, traditional transportation practices negatively affected chicken welfare, correlating with farmers’ lack of awareness and leading to physiological stress responses.

## 1. Introduction

Global trends show that poultry production and consumption are increasing, despite the short-term consequences of the present predicament [1]. The local chicken industry produces the animal protein needs of the Sudanese population. Local chickens are the primary source of protein in food, a significant source of income for people in rural areas, and one of the most effective ways to alleviate poverty [2]. Recently, local chickens have been used in festivals and rituals, in addition to actively assisting with pest management [3]. In Sudan, indigenous chickens make up 80% of the chicken population and are distributed widely throughout communities in urban and rural areas [2]. This is because of their great ability to adapt to the environment and be easily managed, in addition to their contribution in maintaining household food security and generating income [2]. Predators and contagious diseases are reported to be the main threats to villagers’ ability to produce chickens [4].

In contrast to developed countries in the Americas, Europe, and Asia, chicken transportation in the developing world is noticeably different. For example, while animal transportation in developed countries prioritizes meat quality, the transportation of live animals in developing countries, particularly in Africa, often overlooks concerns related to animal welfare [5]. In Sudan’s rural areas, indigenous chickens (Gallus gallus domesticus) are raised in an extensive scavenging free range system. At times, chicken farmers transport their chickens to the city, where a higher price can be obtained. This transportation exposes the chickens to various stressors, including handling, feed withdrawal, loud noises, vibrations, extreme temperatures, social disruption, overcrowding, and limited movement [6]. Stress induced by transportation in chickens is viewed as an adaptive or protective response to safeguard them from the negative impacts of various transportation conditions, including water and feed deprivation, crate density, handling, travel duration, ambient temperature, trailer microclimate, vehicle design, and lairage time [7]. These effects have a direct relationship with the productivity of the birds, the quality of their products, and their welfare. The birds may also experience extreme discomfort, hypothermia, hyperthermia, and a higher chance of suffering serious injury as a result of these conditions. Serum biochemistry, postmortem muscle metabolism, and broiler meat quality are all affected by stress associated with simulated transportation [8]. The transportation of chickens has biochemical, hormonal, physiological, and immunological effects [9,10].

An examination of their blood profile can reveal information about the effects of transportation on poultry and livestock. As immunological mediators, this can be accomplished by measuring the amounts of nutrients, carbon dioxide, oxygen, metabolites, hormones, and heat [11]. Erythrocytes, leukocytes, and thrombocytes are the three cell types that comprise blood. Leukocytes are involved in body immunity, whereas erythrocytes are involved in the exchange and distribution of gases and nutrients within cells, which is crucial for metabolism [12]. Blood glucose levels and the ratio of heterophil–lymphocytes (H/L) are two additional metrics that can be used to evaluate animal welfare in addition to the stress index. The blood profile and stress index may change due to temperature and other physiological conditions [13]. For the majority of mammals, cortisol is the main corticosteroid [14], while corticosterone (CS) is the primary corticosteroid in birds [15]. Activation of the hypothalamus–pituitary–adrenocortical axis typically results in a sudden increase in corticosteroid levels in the blood [16,17]. When corticosterone and other glucocorticoids are produced, the lymphocytes break down, resulting in lymphopenia and ultimately leading the bone marrow to release more heterophils [18]. Plasma glucose levels in commercial layers and broilers [19,20], body core temperature [21], the electrolyte balance, and blood pH [22] have been observed to increase in response to heat stress. All of them could lead to reduced animal welfare and financial loss due to alteration of carcasses [23]. It is well known that reactive oxygen species (ROS) can be generated by stress, which can lead to oxidative damage and a drop in oxidative capability [24]. Oxidative stress can cause damage to cellular molecules such as proteins, DNA, and lipids, as well as tissue injury and cell dysfunction [25]. It has previously been considered that oxidative stress is one of the most vexing difficulties in modern poultry commerce [26]. Chickens under oxidative stress suffer from a number of physiological changes, such as immune system suppression, oxidative damage, and acid-base imbalance. When under stress, chickens try to release heat through evaporative cooling, which causes their respiration rates and rectal temperatures to increase [27].

Most studies have focused on the effects of seasons or distance traveled on domesticated animals’ immunological function, blood and muscle metabolism, stress hormone levels, mortality, and meat quality [28]. However, to the authors’ knowledge, there has been no study describing the negative impacts of the traditional method of transporting birds on the welfare of the indigenous Sudanese chicken, particularly in South Darfur. Therefore, the aim of this study is to evaluate the effect of the traditional transport system on the welfare of indigenous chicken, *Gallus gallus domesticus* in South Darfur state, Sudan.

## 2. Materials and Methods

### 2.1. Questionnaire for Chickens’ Farmers

A total of 42 chicken farmers were interviewed; after explaining the aims of the study, permission was obtained from each chicken farmer. If the farmer was not interested, the opportunity was given to the next interested farmer until we obtained the required sample size. The questionnaire was designed to obtain farmers demographics (sex, age, highest educational level achieved, and job). Furthermore, we aimed to obtain their knowledge about bird welfare and rights, laws/regulations protecting chickens during transportation, and their perceptions towards chicken’s pain/suffering during transportation.

### 2.2. Chickens

**Figure 1:**
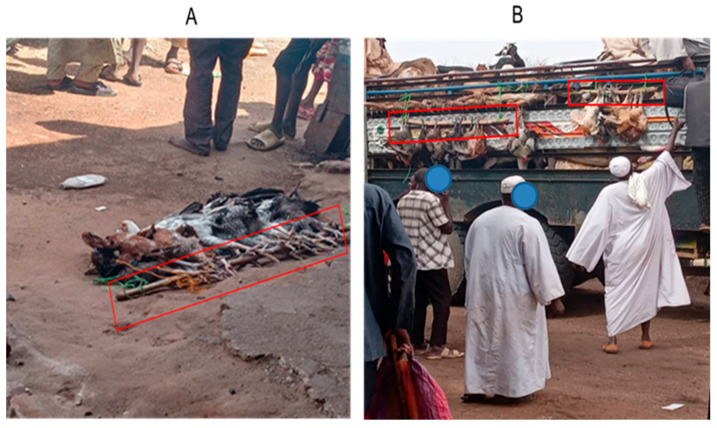
The traditional method of local chicken preparation for transportation. (**A**) Shows the tying of chickens’ legs together on a stick; (**B**) tying the sticks outside the roof the bus.

A total of 160 indigenous (*Gallus gallus domesticus*) chickens (Small Baladi), aged 8 weeks with an average body weight ranging from 700 to 900 g, were obtained from farmers in the market. The chickens were assigned into two experimental groups: a control group and a stressed group, with each group consisting of 80 chickens of equal sex representation. Each group was further divided into eight cages, (*n* = 10 chickens per cage with 3 m^2^ in size). All groups were housed in well-ventilated rooms under standard management conditions, which included unrestricted access to feed and water. The environment maintained a constant temperature of approximately 25 °C and humidity levels around 50%, with a 12 h light-dark cycle throughout the duration of the experiment. The control group chickens were sourced from a chicken market where chickens are regularly purchased. On the other hand, the stressed group chickens were obtained from farmers after they had been transported for 2–3 h from the villages and arrived at the market of the city. For transportation, chicken legs are secured to a stick, which is then suspended in the back or windows of the vehicle (Figure 1). Control group chickens were kept in cage for two weeks for acclimatization. Therefore, the experiment commenced on the first day that the stressed group chickens were bought and continued for 3 sequential days. The tonic immobility test and pecking behavior of two groups for each chicken were recorded daily.

### 2.3. Tonic Immobility Test and Observation of Pecking Behavior

A behavioral test called the tonic immobility test is mostly used to examine stress and fear reactions in birds [29]. Chickens were subjected to the tonic immobility test every day. Each chicken was turned on its side, and a lateral manual restraint was used until the chicken stopped writhing; this was performed to induce tonic immobility. The duration of the tonic immobility was measured from the time the chicken became immobile until it righted itself. If the chicken righted itself in less than ten seconds, the restraining was repeated up to five times. The length of time the bird remains in tonic immobility can be interpreted as an indicator of its fear response. A longer duration may suggest a higher level of fear or stress, while a shorter duration may suggest a lower level of perceived threat or stress. The tonic immobility test was carried out in a separate room inside the same building. A video camera, HWY6 2018 was used to record pecking behavior. It was installed on the wall of the cage and was directed inside, so that the video was recorded for a period of 10 min. The video was watched and the frequency of each chicken that pecked other chickens in the cage was recorded.

### 2.4. Blood Parameters Measurements

Blood samples were collected from chickens by simple venipuncture of the brachial vein on day 2 of the experiment. Sterile 3 mL syringes were used to collect blood. Each blood sample was placed in two tubes; half of blood in a tube containing 0.2 mL of 4% K3 EDTA for leukocytes, and plasma cytokines erythrocytes measurements and other half into a fluoride–EDTA mixture tube for glucose measurement, and then it was mixed gently. Blood profile parameters (total white blood counts (TWBCs), heterophils, lymphocytes, monocytes, basophils and eosinophils), and stress index parameters (heterophil/lymphocyte [H/L] ratio and glucose level) were measured. Red blood cells (RBCs), mean corpuscular volume (MCV), mean corpuscular hemoglobin (MCH), hemoglobin (HB) mean corpuscular hemoglobin concentration (MCHC), and platelet (PLT) were examined based on the method described by Giemsa [13]. The analyses were conducted at the medical Al-ragad laboratory in Nyala city. According to Robertson and Maxwell’s instructions, blood samples were smeared, allowed to air dry, and then stained with May–Grunwald–Giemsa stain in order to determine the leukocytes ratio and differential blood count [30]. A differential blood count was performed under the light microscope and the cells were counted to a total of 100. By dividing the number of heterophils by the number of lymphocytes, the H/L ratio was determined [31].

### 2.5. Pro-Inflammatory Cytokines Measurement

In this study, IL-1β, IL-2, IL-4, IFN-γ, IL-6, IL-17, and TNF-a were tested by using ELISA. We performed the assays based on the instructions of the manufacturer. We seeded the capture antibody of IL-1β, IL-2, IL-4, IFN-γ, IL-6, IL-17, and TNF-a to each well of a 96-well plate overnight. Before streptavidin was added, a second set of biotinylated antibodies was incubated with the plate’s standard antigens the following day. We took a recording at 450 nm when the response color shifted from purple to yellow. For cytokine concentration, the amounts of IL-1β, IL-2, IL-4, IFN-γ, IL-6, IL-17, and TNF-a in each sample were expressed as (ng/L).

### 2.6. Plasma Corticosterone, Total Cholesterol, and Blood Glucose Measurements

Blood for plasma corticosterone (CORT) was collected from the brachial (wing) vein of chickens using sterile syringes. Collections were performed within a defined morning window (08:00–10:00 h) to minimize circadian variation in CORT. A small volume (1.0 mL) was drawn into EDTA-coated tubes. Samples were kept on ice and managed as follows: within 60 min, samples were centrifuged at 12,000× *g* for 10 min at 4 °C to obtain plasma, which was then aliquoted and stored at −20 °C until CORT analysis. We measured plasma corticosterone (CORT) concentrations using an enzyme immunoassay (EIA). We extracted CORT in 5 μL plasma and 195 μL water with 4 mL dichloromethane, re-dissolved in phosphate buffer and triplicated in the enzyme immunoassay. The dilution of the corticosterone antibody (Chemicon, Temecula, CA, USA; cross-reactivity: 11-dehydrocorticosterone 0.35%, cortisol 0.12%, progesterone 0.004%, 18-OHB 0.02%, 18-OH-DOC 0.01%, and aldosterone 0.06%) was 1:8000. We used ABTS [2, 2-azino-bis (3-ethylbenzthiazoline-6-sulphonic acid)] as the substrate and Horseradish peroxidase (1:400,000) linked to CORT functioned as the enzyme label. We used a standard curve that was run twice on each plate to determine the CORT concentrations in plasma. As internal controls, plasma pools from chickens with two distinct corticosterone concentrations were added to each plate. If the concentration was below the detection threshold, we performed the analysis again using 10 μL of plasma. If the concentration was still below the detection threshold, we assigned the value of the lowest quantifiable concentration (1 ngmL-1). We measured the plasma total cholesterol by using commercial assay kits (Nanjing Jiancheng Bioengineering Company, Nanjing, China), based on the manufacturers’ instructions. Furthermore, we used the enzymatic method (GOD-POD) kits, as described previously by [32], to measure the blood glucose concentration.

### 2.7. Oxidative and Antioxidant Indexes

We used the plasma for testing the following enzyme activities, and we used commercial kits to detect the contents. The analysis of malondialdehyde (MDA), catalase (CAT), glutathione peroxidase (GSH Px), and superoxide dismutase, (ATP), and (SOD) were determined by using (PU 8720UV/VIS scanning spectrophotometer, Nanjing, China) in the plasma. We performed all procedures by following the kit’s protocol, and commercial assay kits were delivered by the Nanjing Jiancheng Institute of Bioengineering (Nanjing, China). We performed the examination of ROS by using the OxySelect Assay Kit (STA-San Diego, CA, USA). To measure the amount of ROS in plasma, the highly fluorescent DCF from the oxidation of DCFH by ROS was measured at 480 nm excitation and 530 nm emissions.

### 2.8. Data Analysis

All data of this study were appropriately coded and put into SPSS (Version 21, IBM Crop, New York, NY, USA). A cumulative score was given to each variable, which was a total of their score for each item of the questionnaire. The data analysis focused on generating descriptive statistics (frequencies/percentages) related to the welfare indicators (educational level of the farmers, knowledge of animal welfare and rights, etc.) as obtained through the chicken farmers’ interviews. GraphPad Prism software 9.0 was used to process the clinical tests and behavior observations of the chickens. Mean values ± SEM are used to display the data. Two-tailed Student’s *t* tests were used for statistical analysis. A *p*-value of less than 0.05 was deemed significant.

## 3. Results

### 3.1. Chicken Owner Response to Questionnaire

The results of the participants’ interviews are shown in (Table 1). This study revealed that most of the participants were males. In this study, the majority of the farmers were aged more than 40, 31–40, and 18–30 years old, respectively. In the recent study, the majority of the farmers were uneducated or had reached primary and secondary school, respectively. Furthermore, most of the chicken farmers were seasonal farmers, while others also performed other jobs.

Table 1 shows the indigenous chicken farmers’ interview responses. The results of this study showed that none of the farmers had heard about animal welfare. In this study, when we interviewed the farmers, the majority of them said that they were not aware of animal rights and animal protection laws (69% and 85.7%), respectively. However, the majority of farmers stated that laws protecting animals must be in place before they can be transported from one location to another, while some were unsure if this was a requirement (71.4% and 78.6%), respectively. Almost half of the participants reported not implementing any kind of law to protect chickens while transporting them from place to place, while others implemented them to some extent. The majority of the farmers agreed that chickens feel pain while being transported, while the other farmers indicated that chickens feel pain during transportation to some extent. Moreover, the majority of the farmers indicated that they felt bad about the chickens feeling pain, but a few of them did not care the chickens felt pain during transportation.

### 3.2. Tonic Immobility Test and Pecking

**Figure 2:**
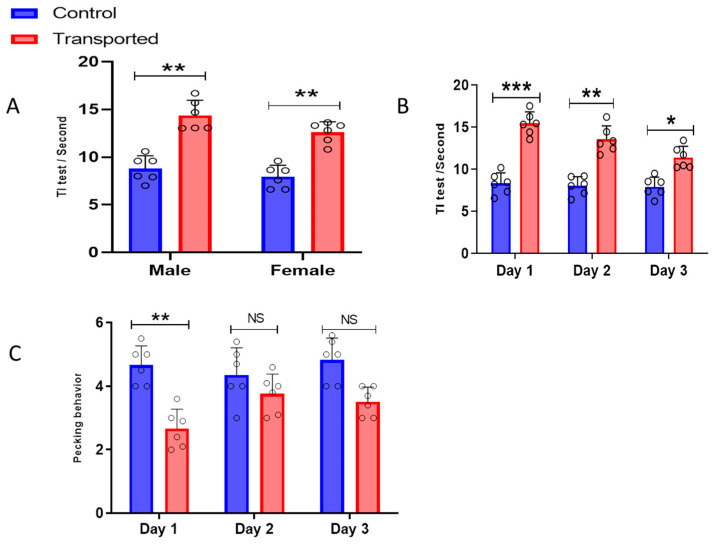
The tonic immobility and pecking behavior results. (**A**) Tonic immobility test results for the control and transported chickens according to chicken sex; (**B**) experimental days; and (**C**) the pecking behavior results for the two groups according to experimental days. Data are presented as means ± SD. * *p* < 0.05, ** *p* < 0.01, *** *p* < 0.001.

The tonic immobility test and pecking behavior results are shown in (Figure 2). Irrespective of sex, tonic immobility time was significantly higher (*p* < 0.05) for transported chickens compared to the controls (Figure 2A). Male transported chickens had a higher tonic immobility duration than females, while no significant difference (*p* > 0.05) between sexes was noted for the control chickens. In the three experimental days, tonic immobility time was significantly higher (*p* < 0.05) for transported chickens than the control group (Figure 2B). Tonic immobility time decreased significantly (*p* < 0.05) from day 1 to day 3 for transported chickens, while no significant difference was observed for the control chickens over time. For both groups, pecking behavior increased from day 1 to day 3 of the experiment (Figure 2C). On day 1, pecking behavior was significantly (*p* < 0.05) higher for the control chickens than the transported group. For day 2 and 3, control chickens had a relatively non-significant (*p* > 0.05) pecking behavior compared to transported chickens (Figure 2C).

### 3.3. Blood Parameter and Pro-Inflammatory Cytokines Measurements

**Figure 3:**
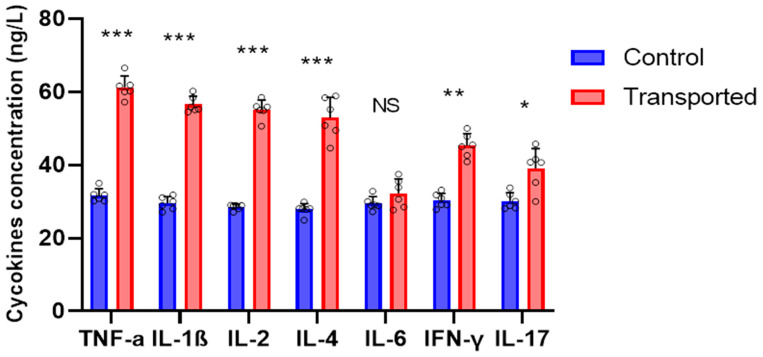
Effects of transportation on the pro-inflammatory cytokines, TNF-α; IL-1β; IL-2; IL-4; IL-6; IFN-γ; and IL-17 concentrations in local control and transported chickens. Data are presented as means ± SD. * *p* < 0.05, ** *p* < 0.01, *** *p* < 0.001.

Table 2 summarizes the mean and range values for the glucose levels, total white blood counts (TWBCs), differential blood counts, and the H/L ratio. Transported chickens had considerably (*p* < 0.05) greater mean levels of glucose, TWBCs, and monocytes than the control group. When compared to the control group, the transferred chickens had considerably (*p* < 0.05) higher mean values for heterophils and the H/L ratio. Furthermore, transported chickens had greater mean levels of basophils and eosinophils than control chickens, although the difference was not statistically significant (*p* > 0.05). Moreover, the result shows that RBCs, Hb, MCHC, and PLT were significantly higher (*p* < 0.05) in transported compared to the control chickens, while no significant difference (*p* > 0.05) was observed in HCT, MCV, and MCH levels between the groups of the chickens (Table 3). There was no significant difference (*p* > 0.05) in IL-6 between the transported and control groups in this study, but the concentrations of TNF-a, IL-1β, IL-2, IL-4, IFN-γ, and IL-17 were considerably (*p* < 0.05) greater in the transported chickens than in the control group (Figure 3).

### 3.4. Oxidative Stress, Corticosterone, Total Cholesterol, and Blood Glucose in the Plasma of the Chickens

**Figure 4:**
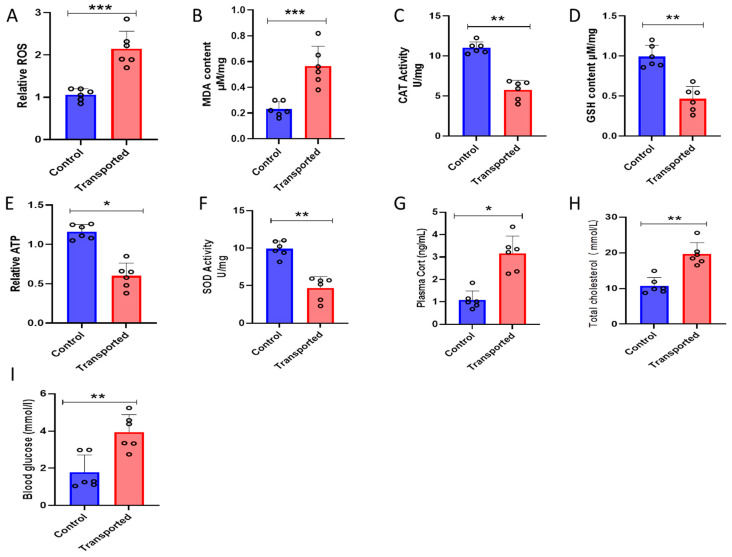
Effects of transportation on the oxidative stress of the local chickens. (**A**) Reactive oxygen species (ROS); (**B**) malondialdehyde (MDA); (**C**) catalase (CAT); (**D**) glutathione peroxidase (GSH); (**E**) the relative parameters of mitochondria ATP; (**F**) superoxide dismutase (SOD); (**G**) plasma corticosterone; (**H**) total cholesterol; and (**I**) blood glucose. Data are presented as means ± SD. * *p* < 0.05, ** *p* < 0.01, *** *p* < 0.001.

As shown in Figure 4, the transported chickens’ plasma ROS and MDA levels were considerably higher (*p* < 0.05) than those of the control group (Figure 4A,B). In contrast, the transferred chickens’ CAT, GSH, ATP, and SOD activities were considerably (*p* < 0.05) lower than those of the control group (Figure 4C–F). Additionally, the transferred chickens had considerably (*p* < 0.05) higher blood glucose, total cholesterol, and plasma Cort than the control group chickens (Figure 4G–I).

## 4. Discussion

The rising number of poultry being raised and the demand for centralized slaughtering in processing plants make transportation a vital component of the poultry industry globally [7]. Depending on the severity of the challenges imposed, every aspect of transportation procedures and practices, as well as the micro-environments present in containers and vehicles, social disruption and noise, fasting and withdrawal of water, may cause the birds to experience varying degrees of stress, compromising their welfare status, health, and productive efficiency [1]. The stress due to chicken transport results in serious weight loss, reduced production performance, increased injury rates, and mass deaths in severe cases [33,34]. Therefore, this study aimed at identifying the welfare issues faced by Sudanese local chickens during traditional transportation. In the present study, most of the participants were male and 18–30 years old. This indicates that a large number of the participants involved in the indigenous chicken industry as a source of income are young males. The study also revealed that the chicken farmers are not knowledgeable about animal welfare, animal rights, and laws protecting the transportation of chickens. Therefore, this suggests that stakeholders of the animal industry, especially the indigenous chicken industry, are very far behind with regard to veterinary extension and animal welfare issues. However, a positive finding is that the majority of them knew that chickens feel pain during transportation and that they feel bad about this issue. This finding suggests that the studied chicken owners are likely to welcome and receive training on animal welfare issues, animal rights, animal transportation laws and guidelines, and other related topics. Local, regional, and international animal welfare organizations can play a critical role in raising the awareness of animal welfare and other related issues to chicken farmer in South Darfur, Sudan.

Behavior is an important indicator of animal welfare. Birds may exhibit normal behaviors such as walking, exploring, fighting, foraging, and decorating in a setting that is naturally stress-free. Behavioral disorders occur when the birds are in a state of stress. As a result of extended stress source activation, poultry behavior is altered from excitation and anxiety to inhibition and depression [35]. Similarly to our study, the tonic immobility time was longer in transported chicken compared to the controls. The chicken pecking behavior was noted to occur at a lower frequency in the transported chickens than the control chickens. Corticosterone and other stress hormones can affect behavior, possibly decreasing energetic or exploratory behaviors like pecking. Additionally, the mobility and strange environment may cause exhaustion and anxiety in transported chickens, which may inhibit their normal actions. The behavior of the chickens may also be impacted by the transportation environment, including variations in temperature, humidity, and air quality [36]. Further studies specifically designed to investigate the relationship between transport stress and pecking behavior would be valuable.

The normal range of the chicken blood glucose level is 230–270 mg dL^−1^ [37]. The results of the present study revealed mean blood glucose levels above normal in transported chicken. The chickens are exposed to stress and do not receive food during transportation. Animal metabolic processes, including chickens, depend on glucose as an energy source and blood glucose levels drop when there is no feeding. Blood glucose levels may also increase if the state of stress continues or if they have chronic stress conditions [13]. Stress stimulates the neurogenic system, which causes the hypothalamus to produce the corticotrophin releasing factor (CRF), which in turn stimulate the anterior pituitary to release the adrenocorticotropic hormone (ACTH) [38,39,40]. Glucocorticoid hormones are secreted by the adrenal cortex tissue cells as a result of ACTH release, which plays a crucial part in gluconeogenesis, a process of creating new sugars from non-glycogen molecules. Proteins and fats are hydrolyzed by glucocorticoids and transformed into either amino acids or fatty acids, respectively, before being transported to the liver. In the liver, fatty acids or amino acids are converted into glucose, which increases blood glucose levels [13].

Heterophils, eosinophils, and mononuclear cells are three types of white blood cells (WBCs) that are typically increased as a result of transport stress in animals [28]. The chickens that were transported had the largest concentration of leukocytes. Due to the high bird density and inadequate air circulation within the vehicle during the three-hour trip, the chickens suffered from increased levels of heat stress and hypoxia during transportation. Chickens produced more leukocytes as a result of this the condition [12].

In reaction to stress, the adrenal glands release glucocorticoid hormones, which cause the bone marrow to produce and release juvenile heterophils, increasing the number of heterophils [41]. The number of heterophils rises as a result of trauma, infection, and exposure to corticosteroids and epinephrine [13]. When compared to control chickens in this study, the transported chickens’ heterophils were higher than usual, which may have been caused by an infection or stress experienced by the birds either before or during the journey. Monocytes are macrophage components that, due to phagocytosing, contribute significantly to the development of non-specific immune responses [42,43]. In this study, the transported chickens had a greater proportion of monocytes than the controls.

Heparin and histamine are both present in the cytoplasmic granules that envelop the nucleus of basophils [44]. Heparin is crucial to keeping blood circulation from freezing. Histamine helps to combat allergies, which contribute to inflammation [45]. The chickens that were transported in this investigation had basophils, whereas the control group had none. But compared to control chickens, transferred chickens had more basophils. The stress of transportation may raise the birds’ basophil numbers, as evidenced by the presence of basophils in the stressed hens and lack thereof in the control group. This may indicate an immunological or inflammatory reaction to the strain of transportation.

Lymphocytes, a subset of leukocytes, mediate a specific immune response by producing antibodies and cellular immunity [46]. When compared to the control group, the mean lymphocyte counts of the transported chickens in the present study were below normal. This outcome is consistent with a prior study [8], which reported a reduction in lymphocytes due to transport stress. A number of physiological and biochemical stress reactions brought on by transportation can be attributed to the variation in mean lymphocyte counts between the transported chickens and the control group. These elements include the effects of stresses in the environment, modifications to metabolic processes, and elevated cortisol levels during transportation. Chickens that are transported have noticeably higher amounts of the stress hormone cortisol, which can inhibit the formation of lymphocytes and the immune system [47]. Transport-related physiological stress can result in elevated heart rate and decreased body weight, which further impairs immunological function. Heat stress, transit distance, and basket density are some of the variables that add to the overall stress that chickens endure, which lowers their lymphocyte counts. Vitamin administration during transit has been shown to preserve physiological characteristics, suggesting that dietary supplements may mitigate some negative effects on lymphocyte counts [48]. It has been proven that the H:L ratio is a stress indicator [49,50]. In chickens, low, optimal, and high stress levels are indicated by H/L ratios of 0.2, 0.5, and 0.8, respectively [31]. In this investigation, the transport chickens’ H/L ratio was greater than that of the control group. This is consistent with earlier research [51,52] which reported increased H/L ratios in chickens exposed to different types of stress.

Higher hemoglobin levels improve cellular function by improving cells’ capacity to transport oxygen and eliminate CO_2_. Hemoglobin levels are influenced by age, species, environment, diet, erythrocyte damage, and handling at the time of examination, according to [53]. As a consequence of transportation stress, the number of erythrocytes caused by hypoxia increased, which is in line with a modest increase in Hb levels in transportation chickens compared to control. The percentage of red blood cells in 100 milliliters of blood is known as the hematocrit value. The greater the hematocrit value, the higher the blood viscosity, which implies that there is more friction between cells [12]. In our study, higher hematocrit values occurred in transported chickens compared to control, but this was statistically insignificant. The capacity of the blood to carry oxygen is significantly impacted by this variation in hematocrit levels [54]. Broiler chickens and other livestock transported at high temperatures will have higher blood viscosity, according to [55]. This slows blood flow in the capillaries and improves the heart. The average hemoglobin concentration in one erythrocyte cell, or the mean ratio of hemoglobin to erythrocyte volume, is known as the mean corpuscular hemoglobin concentration, or MCHC. Transported chickens in this study had high MCHC values which were greater than those shown in control chickens. Broilers generate enormous amounts of red blood cells in the form of reticulocytes, which have a reduced volume during transportation, particularly when exposed to high temperatures and hypoxia [12]. Consequently, hemoglobin is still being produced to create mature erythrocytes, which raises MCHC levels. The chicken eventually reverts to its initial state following transit, hemoglobin production decreases, and MCHC levels decline [13].

Chickens’ physiology and overall health can be greatly impacted by transportation stress, which can change the amount of inflammatory cytokines in their plasma [56]. Many studies have investigated these impacts, emphasizing the intricate relationship between stress, the immune system, and the general health of chickens [57]. Pro-inflammatory cytokines TNF-α, IL-1β, and IL-6 control the host’s immune response and are often elevated in response to stress and disease [58]. In IL-2 and IL-6, it has been shown that pigs’ serum levels of these cytokines rise after transportation stress [59]. Both immunological modulation and macrophage activation depend on the IFN-γ cytokine [60]. In comparison to control hens, the transferred birds in this study showed higher plasma levels of TNF-α, IL-1β, IL-2, IFN-γ, IL-17, and IFN-γ. Studies show that oxidative stress caused by transportation might harm the heart and have other detrimental effects [56] that can significantly impact their physiological state and welfare. Stressor-related variables, adaptation, measurement timing, elevated baseline levels, or a combination of these factors may be responsible for the similar IL-6 levels in the control and stressed chicken groups. However, this resemblance may be because IL-6 can occasionally function as a pro- and pre-inflammatory cytokine. Further investigation into these aspects is needed to fully understand the observed IL-6 response.

Oxidative stress is defined as a physiological stress caused by an imbalance between the generation of ROS due to metal exposure and the antioxidant system’s ability to scavenge ROS [61]. This imbalance results in damage to macromolecules, modifications to the redox state of cells, and disruption of gene expression regulation [62]. SOD activity, which converts O_2_ and H+ into less reactive H_2_O_2_, is the first line of defense for chickens against oxidative stress [63]. Most animals are protected from oxidative stress by CAT and GPH activities, which convert H_2_O_2_ to water and oxygen. CAT also controls the production of ROS within cells, which is related to the control of cellular signaling [64]. In addition, CAT plays a key role in keeping low H_2_O_2_ levels within the normal range, which supports cellular homeostasis and stress adaptation [61]. In the present study, we investigated the levels of several antioxidant-related biomarkers in the plasma to understand the effect of traditional transportation on oxidative stress. The activity of ROS and MDA were increased in transported chickens compared to control. While the activity of CAT, GSH, ATP, and SOD decreased in transported chickens compared to control. However, traditional transportation stress causes oxidative damage to proteins, lipids, and nucleic acids through altering redox dynamics and producing an excessive amount of free radicals.

The hypothalamic–pituitary–adrenal (HPA) axis is activated by transport stress, resulting in plasma corticosterone levels rising quickly. The main glucocorticoid secreted in chickens during stress reactions is corticosterone. According to studies, corticosterone is significantly higher within minutes of acute transport stress (such as in-house handling), reaching its peak 30 to 60 min after the stress [65]. Repeated transport is one example of chronic stress that causes a prolonged rise in corticosterone, which affects immunological and lipid metabolism [66]. Lipid metabolism is directly influenced by corticosterone through upregulating lipogenesis. Long-term exposure to corticosterone raises the expression of lipogenic genes in the liver, leading to accumulation of lipids and an increase in plasma total cholesterol [67]. Stress additionally impacts cholesterol, a lipid involved in many physiological processes, which may have an impact on the general well-being and productivity of chickens [68]. In this study, the traditional transportation of the local chicken elevated the plasma CORT and total cholesterol compared to control chickens, which might lead to overall health problems in the chickens.

The strength of this research is that it is unique and offers a new perspective on the challenges faced by chicken transportation services. Its findings and recommendations intend to help improve the welfare of the chickens during transportation. The limitation of this study was the small sample size, which may impact the generalizability of our results. The sample size was small due to difficulties in recruiting participants and people’s reluctance to take part in research, which was caused by their lack of awareness of the study. Future study with a larger sample is needed to confirm these results and enhance our understanding of the phenomenon under research.

## 5. Conclusions

In conclusion, this study revealed a significant concern regarding the welfare of Sudanese indigenous chickens during transportation, particularly in South Darfur. The findings reveal a clear disconnect between the actual practices employed during chicken transport and farmers’ perceptions of animal welfare. The majority of farmers lack knowledge about animal welfare and rights standards, which directly impacts the comfort of the chickens. The physiological stress indicators observed in transported chickens, such as elevated white blood cell counts and prolonged tonic immobility, underscore the negative effects of current transportation practices. This research highlights the need for increased awareness and education among farmers concerning chicken welfare. Addressing these problems is vital for improving the welfare of indigenous chickens and promoting sustainable farming practices in Sudan.

## Figures and Tables

**Table 1:** Description of the interview responses of the farmers of indigenous chickens (*n* = 42).

Variable	Level	Number	Percentage (%)
Sex	Male	32	76.2
	Female	10	23.8
Age	18–30 years	10	23.8
	31–40 years	16	38.1
	>40 years	16	38.1
Highest educational level achieved	Uneducated	12	28.6
	Primary school	24	57.1
	Secondary school	6	14.3
Job	Farmer	32	76.2
	Teacher	4	9.5
	Other	6	14.3
Did you hear about animal welfare?	Yes	0	0.0
	No	42	100.0
Did you hear about animal rights?	Yes	13	31.0
	No	29	69.0
Do you know animal protection law?	Know	6	14.3
	Do not know	36	85.7
Do you think that transporting animals requires a law to protect them?	Yes	30	71.4
	To some extent	12	28.6
Have you implemented any law to protect chickens during transportation?	Yes	8	19.0
	To some extent	14	33.3
	No	20	47.6
Do you think that chickens feel pain during transportation?	Yes	30	71.4
	To some extent	12	28.6
How do you feel when chickens are in pain?	Feel bad	28	66.7
	Do not care	14	33.3
What is the distance between your area and Nyala city?	51–60 km	14	33.3
	>60 km	28	66.7
How long does it take between your area and Nyala city?	2 h	32	76.2
	3 h	10	23.8
Was the road paved?	Yes	22	52.4
	No	20	47.6

**Table 2:** Mean (range) (TWBCs) levels, differential blood counts, mean, and range of H/L ratio for the control and stress groups.

Variable	Groups	
	Control (*n* = 6)	Transported (*n* = 6)
Mean TWBCs (range)	1716.7 ^a^ (1200–2600)	34,333.3 ^b^ (20,000–40,000)
Mean differential counts (range)		
Heterophils	18.7 ^a^ (10–25)	83.7 ^b^ (70–90)
Lymphocytes	70.0 ^a^ (60–75)	28.2 ^b^ (18–40)
Monocytes	4.8 ^a^ (1–10)	8.8 ^b^ (5–15)
Basophils	0 ^a^	1.5 ^a^ (0–4)
Eosinophils	0.3 ^a^ (0–2)	1.0 ^a^ (1–5)
Mean H/L (range)	0.3 ^a^ (0.1–0.4)	3.2 ^b^ (2.2–4.2)

**Notes:** Significantly different mean values at *p* < 0.05 of group are indicated by different letters in the rows.

**Table 3:** Index of erythrocytes of the control and transported chickens.

Blood Parameters	Control	Transported	*p*-Value
RBC (×10^6^)	2.32 ± 0.07 ^a^	4.70 ± 0.05 ^b^	0.002
Hb (g/dL)	13.56 ± 0.70 ^a^	21.23 ± 0.42 ^b^	0.003
HCT (%)	36.35 ± 1.08 ^a^	39.51 ± 0.75 ^a^	0.076
MCV (fl)	112.86 ± 8.38 ^a^	130.46 ± 5.44 ^a^	0.082
MCHC (%)	39.15 ± 1.73 ^a^	54.67 ± 1.89 ^b^	0.001
MCH (pg)	50.30 ± 2.30 ^a^	59.34 ± 3.30 ^a^	0.061
PLT (×10^8^/L)	0.26 ± 0.02 ^a^	0.554 ± 0.04 ^b^	0.004

**Notes:** Significantly different mean values across two groups at *p* < 0.05 are indicated by different letters in the rows.

## Data Availability

The datasets used and/analyzed in the current study are available from the corresponding author on reasonable request.
